# Endogenous Estradiol and Testosterone may Predispose toward Atherogenic Lipid Profile, but Higher Blood Level of Testosterone is Associated with Lower Number of Stenoses in the Coronary Arteries of Men with Coronary Disease

**Published:** 2006-06

**Authors:** Jerzy Krzysztof Wranicz, Iwona Cygankiewicz, Piotr Kula, Renata Walczak-Jedrzejowska, Jolanta Slowikowska-Hilczer, Krzysztof Kula

**Affiliations:** 1 *Department of Cardiology and Cardiosurgery;*; 2 *Department of Andrology and Reproductive Endocrinology, Medical University of Lodz, Poland*

**Keywords:** coronary stenosis, estradiol, lipid profile, men, testosterone

## Abstract

**Objectives:**

To assess the correlations between blood levels of sex steroid hormones and blood lipid profile or the degree of coronary artery stenosis in men with coronary artery disease (CAD).

**Methods:**

111 men with stable CAD, aged 36-73 yrs, unselected for the coexisting clinical coronary risk factors were prospectively studied. Degree of coronary stenosis was assessed angiographically using different indices. Total cholesterol (T-Ch), high density lipoproteins cholesterol (HDL-Ch), low density lipoproteins cholesterol (LDL-Ch), triglicerydes (TG), testosterone, estradiol, dehydroepiandrosterone sulfate (DHEA-S), and sex hormone binding globulin (SHBG) were measured in the blood. Free testosterone index (FTI) was calculated.

**Results:**

A positive, significant correlations were found between blood concentrations of estradiol and T-Ch (r=0.29, *p*<0.01) or LDL-Ch (r=0.34, *p*<0.005) as well as between FTI and blood LDL-Ch (r=0.23, *p*<0.05). Blood level of estradiol negatively correlated with HDL-Ch/T-Ch ratio (r=-0.21, *p*<0.05). While blood levels of T-Ch correlated positively with 3 out of 5 applied here indices of coronary stenosis, blood LDL-Ch with two of them. In turn, blood level of testosterone negatively correlated with one index of coronary stenosis (r=-0.26, *p*<0.05).

**Conclusion:**

In men with CAD, plasma estradiol concentrations are predictive for T-Ch, LDL-Ch and HDL-Ch/TCh ratio, and FTI for LDL-Ch. Regression analyses indicated that while sex steroid hormones may predispose toward atherogenic lipid profile and are predictive for the number and degree of coronary artery stenosis, higher blood level of total testosterone was associated with the lower number of stenosis in the coronary arteries. Hence, endogenous testosterone may have beneficial effect on coronary arteries.

## INTRODUCTION

Epidemiological studies emphasize the relationship between increased blood level of total cholesterol (T-Ch), low density lipoproteins cholesterol (LDL-Ch) and triglycerides (TG) and the risk of coronary artery disease (CAD) (2, 43). The adult male generally has lower levels of high density lipoprotein cholesterol (HDL-Ch), that protect against arteriosclerosis, and higher levels of LDL-Ch and TG than the premenstrual woman (3). CAD occurs more frequently in men than in premenopausal women (21) and it is hypothesized that this may result from negative influence of androgens and positive of estrogens on the blood lipid profile (2).

In prepubertal children plasma levels of lipoproteins and TG show no gender difference. During puberty plasma levels of HDL-Ch decline in boys, while plasma TG and LDL-Ch increase slightly. Consequently, in men with hypogonadotropic hypogonadism testosterone supplementation to induce puberty leads to an increase in the blood concentrations of T-Ch and LDL-Ch (33). In turn, in older men testosterone administration either decreased (46) or had no influence on T-Ch and LDL-Ch (32). In most studies, however, administration of testosterone in healthy adult men produces a decrease in blood HDL-Ch (4, 32, 46, 53), suggesting negative, unfavorable effect of the testosterone treatment on blood lipid milieu.

In turn, a possible favorable effect of androgens in CAD may be deducted indirectly. For example, a decrease in serum androgen levels was notified in men with acute phase of myocardial infarction (40), however, stress and nonspecific, transient hypogonadism due to acute illness might interfere (42). Lowered blood concentration of testosterone was also seen in men presenting advanced coronary stenosis on angiograms (1, 10, 37). In addition some authors suggest that administration of testosterone may elevate HDL-Ch and decrease TG levels (20, 28, 30).

Relatively recent data indicate a role for estrogen in the male physiology (26, 27). Estrogen receptors (ER) are present in the cardiovascular system and in men with inherited mutation of gene encoding ER (estrogen resistance) the occurrence of premature CAD was documented, suggesting preventive role of estrogens for CAD (44). Consequently, in men with inherited mutation of gene encoding aromatase, an enzyme necessary for estrogen biosynthesis, i.e. in estrogen deficiency increased levels of LDL-Ch and decreased HDL-Ch were found (31). Transdermal supplementation of estradiol in these patients resulted in normalization of lipid profile (8). However, elevated plasma estradiol and estrone have been found in men surviving myocardial infarction, suggesting that hyperestrogenemia may be a coronary risk factor (14).

In the presented here study we aimed to assess if blood levels of endogenous sex steroid hormones (total testosterone, free testosterone index, estradiol and dehydroepiandrosterone sulfate) or sex hormone binding globulin (SHBG) may be predictive for biochemical coronary risk factors (blood lipid profile) or the degree of coronary artery stenosis in a large cohort of male patients with stable CAD in the prospectively projected study.

## MATERIALS AND METHODS

### Patients

111 men aged 36-73 (mean 55 years) with coronarographically documented CAD, that signed informed consent, were invited and prospectively studied. All patients were in stable phase of CAD, 78 patients (90%) had myocardial infarction in the past history (8 to 64 months prior to the study) (I to III stage of CAD according to Canadian Cardiac Society classification). The patients were treated by the following medications at the time of blood drawing: beta-blockers were given to 88% of patients, ACE inhibitors to 73% aspirin in 100%. Patients were not yet treated with statins at the time of blood sampling.

Left ventricle ejection fraction (LVEF) evaluated by two-dimensional echocardiography varied from 20 to 80% (mean 58%) and it was lower in a group of patients after myocardial infarction (20-73%, mean 46%) than in the rest of the patients (LVEF 61-80%, mean 69%). Positive CAD family history (the disease which appeared before the age of 60 in parents and/or siblings) was found in 29 cases. There were 17 active smokers, 28 patients who stopped smoking not longer than 5 years before. There were 41 hypertensive subjects and 14 diabetics. None of the examined men had apparent signs of heart failure. Body mass index (BMI) as well as hip to waist ratio (H/W) were recorded.

### Determinations of blood lipid profile and hormones

For lipids determinations one blood sample was obtained from each patient, in the morning hours, after a 14-hour long fasting. Lipids were assessed by enzymatic method PAP 250 (for T-Ch and TG) and by direct measurement method with BioMerieux kits (for HDL-Ch and LDL-Ch). Fibrinogen level was determined by Clauss method using Multifibren U kit (Dade Behring). As an additional index of atherogenic lipide profile a quotient of HDL-Ch / T-Ch was calculated.

Blood level of estradiol and testosterone were assessed by radioimmunoassay using commercial kits provided by ORION. Dehydroepiandrosterone sulfate (DHEA-S) was determined using Immunotech kits. Sex hormones binding globulin (SHBG) was assayed by radioimmunometric method with commercial DPA kits. To avoid the influence of short-term fluctuations of the hormones in the blood, two blood samples were taken with a 30-min interval (between 7:00 and 8:00 a.m.). These two blood samples were mixed together for a single determination of the hormone in each patient (18). After centrifugation, blood plasma was stored at -20°C, no longer than 60 days, until determinations. To measure the level of unbound testosterone free testosterone index (FTI) was calculated as a quotient of total testosterone level (nmol/l) and SHBG levels (nmol/l).

### Evaluation of coronary artery stenosis

All the patients underwent elective coronary angiography. The percent of stenosis was determined on angiograms by two independent investigators using semiquantative subjective estimation. To measure the extend of coronary artery stenosis 5 different coronary scores were applied. They were based on previously used coronary scores (1, 16, 45). Table 1 demonstrated the way of calculation of the scores. Index A (stenosis score) is a point summary of narrowings in all 15 coronary artery segments (each narrowing was given 1-5 points according to its stenosis degree and then all the points were added). Index B is total number of significant stenosis (over 50% reduction in artery lumen diameter) in all coronary arteries. Index C (vessel score) is the number of the main vessels, including their branches, where the significant changes (over 50% lumen reduction) were found.

**Table 1:** Calculation of coronary artery stenosis by means of coronary indices A, B and C

Degree of stenosis in each of 15 coronary artery segments	Index A Score	Number of stenosis (over 50%) in coronary arteries	Index B Score

none	0	0	0
marginal	1	1	1
0-49%	2	2	2
50-74%	3	3	3
75-99%	4	over 3	4
occlusion	5	diffused non-critical	5
**Index C:** Number of main coronary arteries with their branches, in which critical stenosis was found. Score 1-3			

In addition, two extended indices were elaborated: index A+B and index AxB. They expressed the grade of coronary artery stenosis basing on two parameters simultaneously: the number of the vessels involved and the severity of stenosis in all vessels.

The correlations between the blood levels of hormones and lipid profile and/or the coronary arteries stenosis scores were evaluated using the regression analyses (Spearman’s test). *P* value <0.05 was considered statistically significant.

## RESULTS

Mean ± SD and ranges of different examined parameters are listed in a Table 2. Many of the patients had BMI over 25 kg/m^2^ and all had the increased values of H/W ratio, indicating increased abdomen fat accumulation, an important risk factor for arteriosclerosis. 52 out of 81 patients (65%) were overweight.

**Table 2:** Mean values, standard deviation and ranges of the examined variables

Variables	Mean value ± SD	Minimum	Maximum

Age	55.2 ± 8.8	36.0	73.0
BMI [kg/m^2^]	27 ± 3.0	18.6	31.1
H/W	1.02 ± 0.07	0.94	1.2
TCh [mg/dl]	229.4 ± 44.6	144.0	355.0
HDL-Ch [mg/dl]	41.9 ± 12.8	19.0	85.0
TG [mg/dl]	160.7 ± 63.9	53.0	338.0
LDL-Ch [mg/dl]	157.4 ± 38.6	74.0	248.0
HDL/TCh	18.4 ± 5.6	0.5	37.3
Fibrinogen [mg/dl]	338.0 ± 4.6	195.0	540.0
Testosterone [nmol/l]	18.7 ± 7.1	6.8	39.0
SHBG [nmol/l]	58.0 ± 31.0	17.0	164.4
FTI	0.4 ± 0.2	0.1	1.0
DHEA-S [mg/ml]	139.0 ± 87.0	23.6	404.9
Estradiol [pmol/l]	5.1 ± 3.1	1.0	18.2

SD, standard deviation; BMI, body mass index; H/W, hip/waist ratio; TCh - total cholesterol; HDL-Ch, HDL-cholesterol; TG, triglicerydes; LDL-Ch, LDL-cholesterol; SHBG, sex hormone binding globulin; FTI, free testosterone index; DHEA-S, dehydroepiandrosterone sulfate.

Blood plasma concentration of total testosterone ranged from hypogonadic level (6.8 nmol/l) to values exceeding the norm (39.0 nmol/l) (normal values 8-34 nmol/l). Great variability concerned also SHBG, DHEA-S and estradiol as well as the calculated FTI.

**Figure 1:**
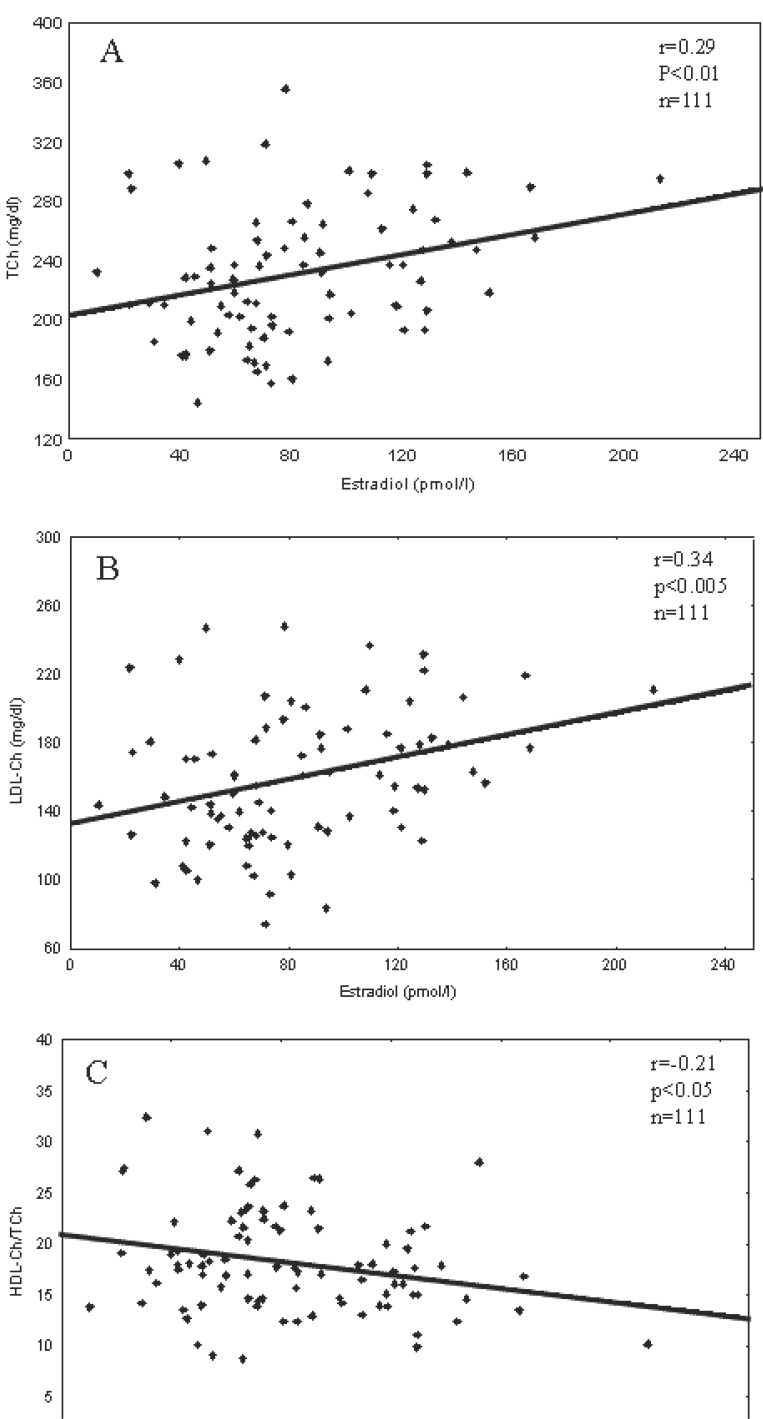
Correlation between blood plasma concentration of estradiol and concentrations of total cholesterol (TCh) (A), LDL-cholesterol (LDL-Ch) (B) or HDL-Ch/TCh ratio (C) (Spearman’s test).

**Figure 2:**
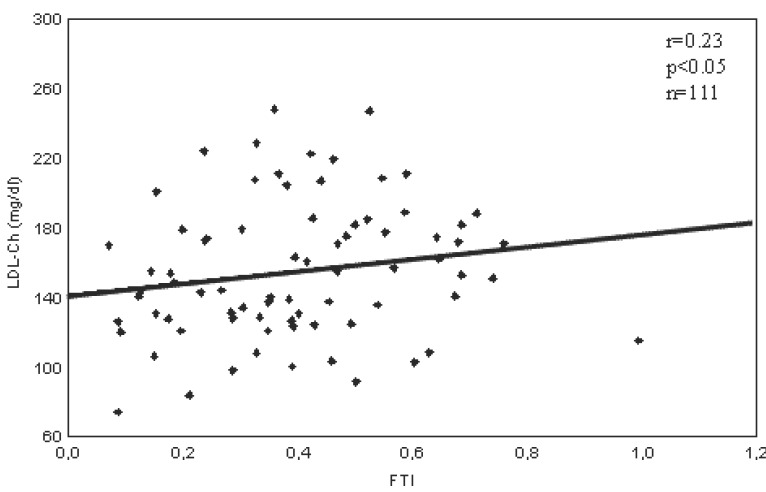
Correlation between free testosterone index (FTI) and blood concentration of LDL-cholesterol (LDL-Ch) (Spearman’s test).

The significant positive correlations were found between blood level of estradiol and T-Ch or LDL-Ch and negative between estradiol and HDL-Ch/TCh ratio (Fig. 1). FTI positively correlated with LDL-Ch (Fig. 2). Blood level of T-Ch positively correlates with the index A of coronary artery stenosis, while both T-Ch and LDL-Ch positively correlated with the values of the extended indices (A+B or AxB). Blood total testosterone positively correlated with the index B (Table 3).

**Table 3:** Correlations between different indices of coronary artery stenosis (A, B, C, A+B, and A × B) and the levels of blood lipids, sex steroid hormones, sex hormone binding globulin (SHBG) or free testosterone index (FTI). See explanations of the meanings of A, B and C in Table 1

		Indexes of the stenosis of coronary arteries				
		A	B	C	A + B	A × B

TCh	r	0.26	0.23	0.18	0.29	0.31
	p	<0.05	NS	NS	<0.01	<0.005
LDL-Ch	r	0.20	0.17	0.16	0.25	0.27
	p	NS	NS	NS	<0.05	<0.05
HDL-Ch	r	-0.04	0.16	-0.08	0.01	-0.02
	p	NS	NS	NS	NS	NS
Total testosterone	r	-0.01	-0.26	-0.01	-0.08	-0.01
	p	NS	<0.05	NS	NS	NS
DHEA-S	r	-0.07	0.06	-0.02	-0.04	-0.01
	p	NS	NS	NS	NS	NS
Estradiol	r	-0.01	-0.13	-0.01	-0.05	-0.04
	p	NS	NS	NS	NS	NS
SHBG	r	0.05	0.09	0.03	0.07	0.04
	p	NS	NS	NS	NS	NS
FTI	r	0.04	0.03	0.12	-0.03	0.82
	p	NS	NS	NS	NS	NS

r, coefficient of correlation (Spearman’s test); NS, non significant.

While blood levels of T-Ch correlated positively with 3 out of 5 applied here indices (A, A+B and AxB), blood LDL-Ch correlated with two of them (A+B and AxB). In turn, blood level of testosterone negatively correlated with one index B of coronary stenosis (r=-0.26, *p*<0.05), reflecting the number of stenosis. No other significant correlations were present.

## DISCUSSIONS

In our previous report (50) we already demonstrated that in men with CAD blood level of estradiol correlated positively with blood level of T-Ch and LDL-Ch, however, our present results extended greatly those reported previously, because we have shown here that: 1) In men with CAD, blood concentrations of T-Ch, LDL-Ch and total testosterone are predictive for the degree of coronary artery stenosis and hence, these biochemical parameters are sensitive measures of CAD; 2) Blood concentrations of estradiol are predictive for the levels of T-Ch, LDL-Ch and HDL/T-Ch ratio and FTI is predictive for LDL-Ch. It suggests that both, endogenous estradiol and testosterone, may predispose toward atherogenic lipid profile; 3) Regression analyses indicated, however, that endogenous testosterone may be beneficial for male health promotion as concern the number of coronary artery stenosis.

The finding that estradiol may predispose toward atherogenic lipid profile in men with CAD may support also the earlier data demonstrating that blood level of estradiol positively correlated either with T-Ch (11, 23) or LDL-Ch (22) in different populations of men with CAD. In addition we have shown that estradiol negatively correlated with HDL-Ch/T-Ch ratio, which is the most discriminative index for the atherogenic lipid milieu and the higher HDL-Ch/T-Ch ratio, the better for the patient (39). The associations of endogenous estradiol with T-Ch or LDL-Ch may not apply to postmenopausal women treated with exogenous estrogen, where an increase in HDL-Ch and the reduced blood level of T-Ch and LDL-Ch were noticed (12, 24, 29).

In the human male, hyperestrogenemia per se was suggested to be an independent coronary risk factor, when significantly elevated estradiol levels were found in men surviving myocardial infarction (14, 30, 34, 38). In patients treated with estrogens for prostate cancer, an increase in cardiovascular death, fatal stroke (47) and nonfatal myocardial infarction (11) were noted. In this context it has been also shown that blood level of estradiol positively correlated with the advance of atherosclerosis, examined by angiography (17, 30) and with the indices of blood coagulation (38), the findings that were not supported by our study.

The Coronary Drug Project (11), begun in the 1960’s, was the first trial designed to determine whether estrogen reduced the risk of CAD. Men with known heart disease were randomly assigned to one of five active therapies or placebo. The estrogen arms of the study were stopped because estrogen-treated men had an increased rate of thromboembolic events and myocardial infarction. After the findings that estrogen failed to protect men or women, no large trials with estrogen treatment of CAD were initiated in man.

We demonstrate here a positive correlation between FTI and blood LDL-Ch but not between total testosterone and blood lipids. FTI is an index of biologically active testosterone, not conjugated with blood proteins. The binding protein for testosterone is predominantly SHBG. Production of SHBG in the liver may reveal individual variation and significantly influence peripheral bioavailability of testosterone. Therefore, an assessment of SHBG and calculation of FTI permit to evaluate indirectly bioavailable testosterone and is highly reliable (48). The positive correlation between FTI and LDL-Ch blood levels suggests that endogenous testosterone may promote the development of atherogenic lipid profile. However, in some previously published data blood testosterone negatively correlated with LDL-Ch or T-Ch and positively with HDL-Ch (7, 13, 15, 23, 37, 52). Most of the previously published studies were, however, based on a single blood sampling and usually long lasting (years) storage of the samples. Because steroid hormone concentrations undergo substantial daily fluctuation (9), hormone measurements were made here on a double plasma sampling. It has been suggested that estradiol values decrease with the time in storage, even at -70C (9, 35, 36). Here, the samples were stored no longer that 60 days, so no sample deterioration was possible. The additional advantage of our study was a hormone measurement procedure that involved assays run on single commercial immunoassay kits that eliminated inter-assay variations.

Our data may be in accordance with those presented by other authors (although FTI was not calculated) showing that in men with hypogonadism testosterone supplementation leads to an unfavorable increase in the blood concentration of T-Ch and LDL-Ch (33). Excessive testosterone exposure in men is uncommon in clinical practice. However, anabolic-androgen steroid (AAS) abuse is very popular. In the literature covering 12-yr period from 1987-1998, there was a total of 17 case reports of cardiovascular events in young male body builders using suprapharmacological doses of AAS. It has been suggested that dose-dependent androgen-induced vasospasm, platelet aggregation, activation of coagulation cascade, atherogenic lipid profiles (increased LDL-Ch and decreased HDL-Ch), and abnormal left ventricular function and hypertrophy are relevant mechanisms precipitating sudden cardiac deaths in young power athletes and body builders (for review see 51).

However, our results support those suggesting that the lower the androgens in the blood, the higher the coronary stenosis score (1, 10, 37), indicating a plausible, positive influence of testosterone on the coronary arteries. The negative correlation of total testosterone with index B suggests that testosterone may be beneficial for coronary arteries and that higher blood levels of testosterone is associated with the lower number of stenosis over 50% of the lumen. Higher DHEA-S concentrations were reported earlier to be associated with lower degree of the stenosis of coronary arteries and better lipid profile (1, 5, 19, 49), however, it was not supported by our findings. These discrepancies may arise from differences in precision of the subjective measurement of the coronary artery stenosis scores in different centers and in other methodological approaches. For example, while here the prospective cohort study was conducted on the ambulatory basis, in other centers retrospective studies, based on consecutive hospitalized patients, were conducted.

Beside the postulated here promotion of the development of atherogenic lipid milieu by estrogen and androgen, possibly through their influence on hepatic lipase activity, plasma levels of sex steroid hormones may also reflect changes in blood lipids due to mechanistic relationships between biochemical precursors and products. Namely, LDL-Ch is a fraction of cholesterol utilized for the biosynthesis of sex steroid hormones in Leydig cells (25). Therefore, the changes in plasma concentrations of LDL-Ch, serving as a substrate, may be followed by the changes in biosynthesis of sex steroid hormones, that are products of LDL-Ch bioconversion. The observed here similarity between two circulating sex steroids (estrogen and bioavailable testosterone) in relation to blood lipids may not be surprising when considering that testosterone is a prehormone for estradiol and that many effects of testosterone in men is facilitated through estradiol. Local modulation of the balance of androgen/estrogen action could be envisaged to regulate target cell function. In this sense neither androgen nor estradiol alone but androgenic and estrogenic branches of the sex steroid pathway could be involved in physiology and pathology (41).

Summing up, our results suggest that in the human male the increased levels of both endogenous estradiol and testosterone may predispose toward atherogenic lipid profile which in turn predisposes toward coronary artery stenosis. However, endogenous testosterone may be beneficial for coronary arteries as we have found that higher blood level of total testosterone was associated with the lower number of stenosis in the coronary arteries.
